# Circadian rhythm entrainment of the jewel wasp, *Nasonia vitripennis*, by antagonistic interactions of multiple spectral inputs

**DOI:** 10.1098/rspb.2022.2319

**Published:** 2023-02-08

**Authors:** Yifan Wang, Gregor Belušič, Ido Pen, Leo W. Beukeboom, Bregje Wertheim, Doekele G. Stavenga, Roelof A. Hut

**Affiliations:** ^1^ Groningen Institute for Evolutionary Life Sciences, University of Groningen, 9712 CP Groningen, the Netherlands; ^2^ Department of Biology, Biotechnical Faculty, University of Ljubljana, 1000 Ljubljana, Slovenia

**Keywords:** action spectrum, phase shift, light, colour opponency, photoreceptor, biological clock

## Abstract

Circadian light entrainment in some insects is regulated by blue-light-sensitive cryptochrome (CRY) protein that is expressed in the clock neurons, but this is not the case in hymenopterans. The hymenopteran clock does contain CRY, but it appears to be light-insensitive. Therefore, we investigated the role of retinal photoreceptors in the photic entrainment of the jewel wasp *Nasonia vitripennis*. Application of monochromatic light stimuli at different light intensities caused phase shifts in the wasp's circadian activity from which an action spectrum with three distinct peaks was derived. Electrophysiological recordings from the compound eyes and ocelli revealed the presence of three photoreceptor classes, with peak sensitivities at 340 nm (ultraviolet), 450 nm (blue) and 530 nm (green). An additional photoreceptor class in the ocelli with sensitivity maximum at 560–580 nm (red) was found. Whereas a simple sum of photoreceptor spectral sensitivities could not explain the action spectrum of the circadian phase shifts, modelling of the action spectrum indicates antagonistic interactions between pairs of spectral photoreceptors, residing in the compound eyes and the ocelli. Our findings imply that the photic entrainment mechanism in *N. vitripennis* encompasses the neural pathways for measuring the absolute luminance as well as the circuits mediating colour opponency.

## 1. Introduction

In almost all organisms, the circadian clock system is a crucial endogenous mechanism, regulating orientation, foraging and anticipation of environmental changes [1]. Adaptation to rapid environmental changes may therefore depend on the flexibility of circadian clock systems. This is particularly true for invertebrates that possess less elaborate physiological mechanisms to buffer against environmental disturbances than warm-blooded animals, such as birds and mammals.

Circadian photoreceptors have been well studied in the model insect, *Drosophila melanogaster*. The blue-light-sensitive protein cryptochrome (CRY) was first identified as the main circadian photoreceptor for the entrainment in *Drosophila* [2]. CRY is a deep-brain circadian photoreceptor pigment that is expressed in a specific subset of *Drosophila*'s clock neurons as well as in the compound eyes [3,4]. In *Drosophila*, multiple classes of photoreceptor cells have been identified as circadian photoreceptors, which are located in the compound eyes, the dorsal ocelli, the extra-retinal Hofbauer–Buchner eyelet and Bolwig's organs [5]. Their light sensitivity is due to visual pigments, the rhodopsins [6].

For most insect species, the photoreceptors mediating the photoperiodic rhythms remain unclear. In the cockroaches, *Leucophaea maderae* and *Periplaneta americana* and the cricket *Gryllus bimaculatus*, compound eyes have been identified as the only photoreceptive organ for circadian entrainment [7–10]. In *G. bimaculatus*, the green-sensitive photoreceptor class of the compound eyes is reported as the major circadian photoreceptor, but additionally a novel photic molecular entrainment pathway has been proposed [11,12]. In other cricket species, *Teleogryllus commodus*, *Acheta domesticus* and *Dianemobius nigrofasciatus*, besides the compound eyes, also the ocelli, so-called simple eyes, are involved in the circadian regulation [10,13,14]. In beetles, which generally lack both light-sensitive CRY and ocelli, circadian entrainment is presumably mediated through the compound eyes and the lamina organs or the stemmata [15]. Honeybees (*Apis mellifera*) appear to possess a deep neuronal photoreceptor, containing pteropsin, that may be involved in circadian entrainment [16]. Like other Hymenoptera, the compound eyes of honeybees contain three visual photoreceptor classes, with UV-, blue- and green-sensitive rhodopsins [17], but a possible role of those photoreceptors in circadian photoreception has not yet been established.

To gain a mechanistic understanding of the circadian system and entrainment pathways in Hymenoptera, we aimed at identifying the circadian photoreceptors and their spectral sensitivities in the jewel wasp *Nasonia vitripennis*. *N. vitripennis* is a small solitary wasp that has been extensively used to study circadian rhythms and insect seasonality. It is diurnally active and exhibits a distinct photoperiodic response to shortening of the photoperiod [18,19]. It is parasitic on flies and features a robust circadian rhythm in its locomotor activity upon emergence from the host pupa and in courtship, with a strong light-resetting mechanism [20–23]. It also exhibits a high sensitivity to photoperiod change for diapause induction [24,25]. The light-dependent diapause response regulation is most sensitive to longer wavelengths (554–586 nm), but the spectral sensitivity varies between dawn and dusk [26,27].

The specific aim of the present study is to identify the photoreceptors providing circadian input to *Nasonia*. The chosen circadian behavioural response was the light-induced phase shift in the locomotor activity of the wasps in response to a light pulse of a particular wavelength and light intensity. An action spectrum for the light-induced circadian phase shift can thus be obtained.

As for potential photopigment candidates, there are four opsin genes coding for the photosensitive rhodopsins of *Nasonia* according to genome annotation [28]. Because the exact spectral characteristics and expression patterns of *Nasonia*'s rhodopsins are unknown, we performed electrophysiological recordings in the compound eyes and in the ocelli. The combined electrophysiological and behavioural results indicate a central role of the photoreceptors in the compound eyes as well as the ocelli for the circadian photoentrainment in *N. vitripennis*.

## 2. Materials and methods

### (a) Experimental lines and maintenance

The experiments were performed with the standard *Nasonia vitripennis* laboratory strain AsymC. This is the LabII wild-type strain from Leiden, The Netherlands, cured of its *Wolbachia* infection with antibiotics, and maintained in the laboratory since 1971 [29,30]. The whole genome of this strain has been sequenced and annotated [28]. *N. vitripennis* is maintained as a mass-culture in a temperature and humidity-controlled incubator (20 ± 1°C, 50–55% RH) under a light–dark cycle of 18 L : 6 D (646 lux) to prevent diapause induction and maintain a 21-day generation time. Wasps were kept in plastic vials (70 × 20 mm) with *Calliphora* spp. fly pupae as hosts, providing approximately 15–20 females with 30–50 fly hosts each generation. The experimental wasps, 1- to 2-day-old-mated females, were individually placed in cotton-plugged polystyrene tubes (60 × 10 mm) and supplied with three host pupae for optimal development of their offspring.

### (b) Locomotor activity measurements

For all locomotor activity recordings, unmated females at the black pupal stage (approx. 17–18 days after eggs were laid) were collected and put into cotton-plugged polystyrene tubes with a piece of filter paper dipped in sugar water. After eclosion, 1- to 2-day-old virgin females were transferred into locomotor activity tubes (65 × 5 mm) that were for one quarter filled with agar food (30% sucrose, 1.5% agar, 0.1% nipagin) and closed with a black plastic plug on both ends. Individual locomotor activity was recorded by the Drosophila Activity Monitoring System (DAMS, TriKinetics, Waltham, USA) with 32 tubes in one monitor in each experimental condition every minute, by infrared light beam break at the middle of the tube. The monitors were placed into light-tight boxes (23 × 14 × 32 cm^3^) in a temperature and humidity-controlled climate room (18 ± 1°C, 50–55% RH). We used slightly lowered temperatures in the experiments to prolong their lifespans. In total, 12 light-tight boxes were used simultaneously for the experiments. For determining the optimal settings for the phase shift experiments, each light-tight box was fitted with a LED light source (ILH-GD01-NUWH-SC201, Neutral White 4000 K, PowerStar, Berkshire, UK) covered by a light-diffuser sheet (electronic supplementary material, figure S1; electronic supplementary material, SI).

### (c) Circadian action spectrum measurements

When investigating the effects of light on the circadian rhythm, there are at least four important factors of the stimulus that need consideration: its timing, duration, intensity and spectral composition. In the action spectrum experiments, the light intensity and spectral composition of the stimulus were varied, while the timing and duration were kept constant. To determine the optimal entrainment light–dark cycle, wasps were first entrained under light–dark cycles ranging from 4 L : 20 D to 18 L : 6 D for a period of 7 consecutive days, and subsequently left free-running in complete darkness (DD) for another 10 days. The sample size for each condition was between 10 and 20 animals. From this pilot experiment, the 14 L : 10 D cycle was determined as the most optimal entrainment cycle.

Phase response curves were then measured to determine the timing of the maximal circadian light response after a single light pulse, following the Aschoff type II protocol [31]. All animals in the 12 light boxes were entrained under the same light–dark cycle of 14 L : 10 D during the first 7 days. On day 8, individuals in each light box received a 6 h light pulse at 12 different time points, respectively from *Zeitgeber* Time ZT0 to ZT22 in 2 h intervals, followed by constant darkness (DD) for another 10 days. One dark control group was also tested under the same conditions on day 8, but without any light pulse, to establish the phase angle of entrainment without additional light-induced phase shift. The sample size for each condition group was between 10 and 20 wasps.

For the action spectrum experiments, wasps were entrained in the same light-tight boxes as before, for the first 7 consecutive days under the light–dark cycle of 14 L : 10 D. At ZT18 on day 8, a 6 h monochromatic light stimulus was given to the test animals in a different set-up, the light stimulator box (53 × 53 × 117 cm^3^), in which monochromatic light was provided by a halogen light source equipped with a light-diffuser sheet and one of a series of monochromatic light filters (electronic supplementary material, figure S1). Four small boxes (22 × 21 × 29 cm^3^) were placed below the light stimulator box, each equipped with a different layer of neutral density filters (LEE Neutral Density filters 0.5–1.2 ND) to control light intensity, and in each of the four boxes, a DAM monitor containing 32 wasps was transferred. This light stimulator set-up allowed one specific wavelength and four different light intensities to be tested at the same time. To detect the new phase, after the light stimulus was given, DAM monitors were placed at the light-tight boxes, which were kept under DD conditions for another 10 days. All transfers of the activity monitors containing the wasps were done in complete darkness inside the climate room. In total, for each condition 14 different wavelengths were tested with a sample size of 12–38 wasps; the number depending largely on mortality.

### (d) Determination of phase shifts

Phase shifts were analysed in ChronoShop [32], by taking into account the changes in circadian period before and after the light pulse, using Sokolove and Bushell periodogram analysis [33]. The first two transient days after the light pulse were excluded in all analyses and the centre point of gravity was used as the phase marker [34]. The ChronoShop program is an automated method of calculating phase shift, developed to detect proper phase markers in an objective way, and to calculate changes after a light pulse based on all circadian cycles. The ChronoShop program calculates the circadian phases for each cycle by taking into account of the average activity patterns prior or after the light pulse and using forward/backward extrapolation. Regression lines are then plotted through the phase markers to calculate the actual phase shift—the differences between the phase markers of the two phases at the same time of the light pulse. Individuals that showed early death or arrhythmic activity patterns were excluded from the analysis. The average dark control phase shift was subtracted from phase shifts after light stimulation. Average phase shifts were calculated using a circular average on a 24 h scale.

### (e) Statistical analysis

The data were analysed and plotted in Matlab (R2021a) or R (v. 4.1.2). For analysing the action spectrum data, we used a generalized additive model (GAM) to derive the effects of light intensity and wavelength on the phase shift responses, as GAM is useful to fit nonlinear curves in the absence of a theoretically well-defined nonlinear function. In the applied GAM model, the phase shift was defined as response variable, while wavelength and log-light intensity were defined as predictor variables, using the gam function of the mgcv R package [35]. We used mgcv's default thin-plate splines with 10 knots each (gam arguments: bs = ‘tp’, k = 10). Restricted maximum likelihood (gam argument: method = ‘REML’) was used to fit the final model, while maximum likelihood (gam argument: method = ‘ML’) was used when comparing different models with likelihood ratio F tests (mgcv function anova). Splines for both predictors were combined additively, and interactions between predictors were fitted with tensor splines (mgcv function ti). Significance of interactions was tested by comparison of models with and without an interaction term.

### (f) Photoreceptor intracellular recordings and electroretinogram measurements

The spectral sensitivity of the photoreceptors in the compound eyes and ocelli of *N. vitripennis* was determined with intracellular (single-cell) and extracellular recordings (electroretinogram (ERG) with light adaptation). Electrophysiological measurements were performed in wild-type (AsymC) strains. The wasps were immobilized using bee wax and resin and pre-oriented on a mini-goniometer, mounted on a larger, rotatable goniometric stage that carried a micromanipulator and microelectrode (Sensapex, Oulu, Finland). The goniometer was rotated during the recordings with respect to the light stimulus to yield maximal light responses. The electrodes were pulled from borosilicate glass on a horizontal puller (P-2000, Sutter, Novato, CA, USA). For extracellular recordings, electrodes with a 1–5 µm tip were filled with insect saline (0.67% NaCl, 0.015% KCl, 0.012% CaCl_2_, 0.015% NaHCO_3_, pH 7.2) and inserted just below the cornea of the compound eye or into the head capsule next to individual ocelli. For intracellular recordings, a microelectrode filled with 3 M KCl and resistance 80–120 MΩ was advanced into the retina through a small hole at the edge of the cornea. The reference electrode was a 50 µm diameter Ag/AgCl wire, inserted into the thorax. The signal was amplified with a SEC 10 LX amplifier (npi electronic, Tamm, Germany), conditioned with a Cyber Amp 320 (Axon Instruments, Union City, CA, USA) and digitized via a Micro1401 (CED, Cambridge, UK) analogue–digital (A/D) converter.

Spectral stimulation was provided with an array of LEDs, combined with a diffraction grating (‘LED synth’ [36]), or with light from an XBO arc lamp (Cairn, Faversham, UK), filtered with a monochromator (B&M, Limburg, Germany) at 10 nm bandwidth. Both light sources were tuned to emit equal numbers of photons at any wavelength (‘isoquantal’ mode) and were projected on the eye coaxially with the recorded photoreceptor. The aperture of the stimulating beam was adjusted with an iris diaphragm. The spectral sensitivity measurements were performed either in the dark-adapted eye or with the eye selectively adapted to constant light from the LED synth; both light sources had a wide aperture (approx. 20°). For intracellular recordings, the stimulating aperture was kept at approximately 2° to minimize ERG artefacts. The response amplitudes of single cells were transformed to sensitivities by means of an intensity–response function and the reverse Hill transformation [37]. The obtained spectral sensitivities were fitted with single rhodopsin templates or a weighted sum of three rhodopsin templates. Invertebrate-specific visual pigment templates were used to calculate the absorption spectrum of *Nasonia* rhodopsins [38].

## 3. Results

### (a) Phase response curves and circadian action spectrum

**Figure 1.**
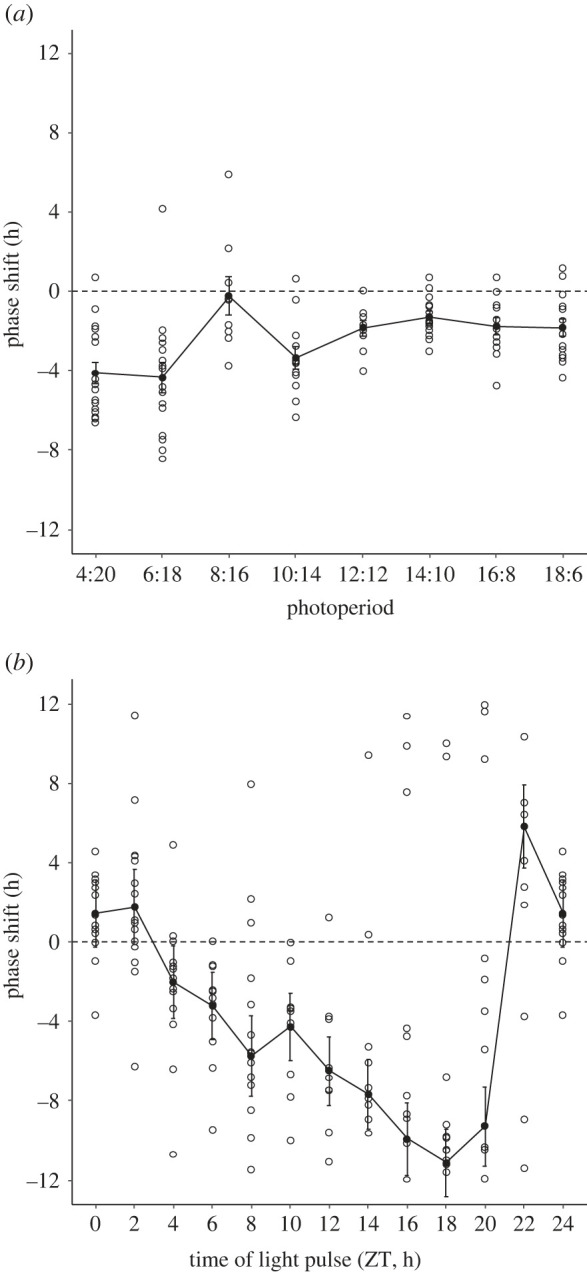
Phase shift responses of *Nasonia vitripennis* under different photoperiod and with light stimulation at different time points. (*a*) Phase shift in the dark: entrainment for 7 days under different photoperiods (L : D) and then free-running in darkness. (*b*) Light-induced phase shift: entrainment for 7 days under a 14 L : 10 D photoperiod followed by a 6 h white light stimulation on day 8 and then free-running in darkness (black dots: average phase shifts; error bars: s.e.).

We determined the optimal entrainment light–dark cycle of *Nasonia's* light-dependent phase shift response by entraining wasps for 7 days under light–dark cycles ranging from 4 L : 20 D to 18 L : 6 D, followed by measuring free-running in complete darkness (DD) for another 10 days. The lowest inter-individual variation in circadian phase was found after 7 days of 14 L : 10 D cycle, meaning that we could construct the circadian action spectrum with the smallest sample size under this condition (figure 1*a*). Thus, the 14 L : 10 D cycle was determined as the optimal entrainment cycle. Additionally, since the wasps experienced no light pulse in this protocol, they also served as the control group. Previous research demonstrated that light intensity and duration could induce different levels of phase shift response in *Nasonia*, resulting in either a weak phase response curve (type 1 PRC) or a strong type 0 PRC [23]. To avoid the ambiguous transition between type 1 to type 0 phase shift response, we gave a 6 h lasting, high-intensity white light stimulus. A maximum phase shift of nearly 12 h phase delay occurred when the light pulse was given at ZT18 (figure 1*b*).

**Figure 2.**
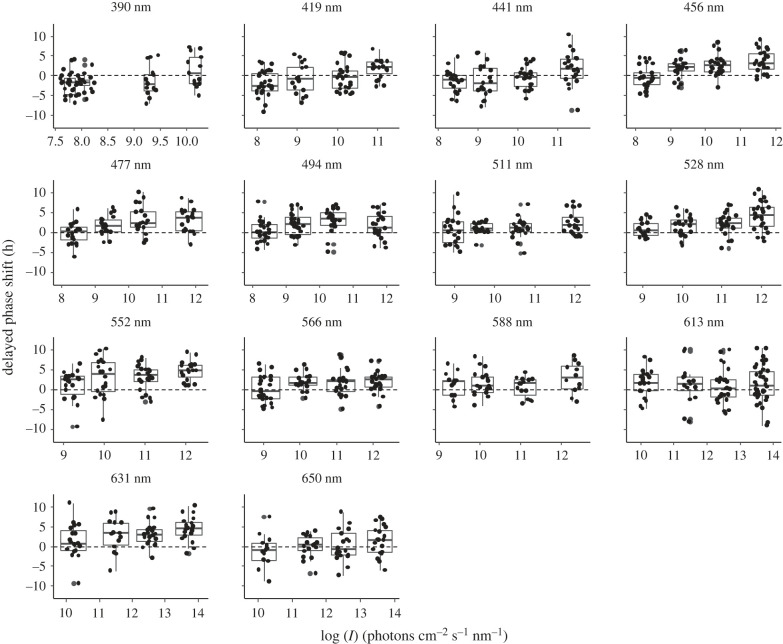
Phase shift responses of *Nasonia vitripennis* to light stimuli with different wavelength and intensity. Delayed phase shift is plotted here since the majority of the phase shift responses were delayed, making it easier to see increased phase responses with increased light intensities. Boxes represent the delayed phase shift from the first quartile to the third quartile. The horizontal line in each box is at the median of delayed phase shift; the lower vertical lines go from the first quartile to the minimum and the upper vertical lines go from the third quartile to the maximum of the delayed phase shift.

To construct the circadian action spectrum of the phase shift response, all animals were entrained under a 14 L : 10 D photoperiod for 7 days. On day 8 at ZT18, monochromatic light stimuli were given at 14 different wavelengths and with four different intensities per wavelength (illumination spectra in electronic supplementary material, figure S1). Light stimulation elicited phase shift responses that generally increased with increasing light intensity. Since the light stimulation was given in the middle of the subjective night, the majority of the phase shift responses were delayed, and we therefore analysed the data as delayed phase shift. The phase shift responses varied between 12 h delay and nearly 10 h advance (figure 2). This kind of high variations in circadian responses is consistent with previous reports on *Nasonia*'s circadian behaviours [23].

**Figure 3.**
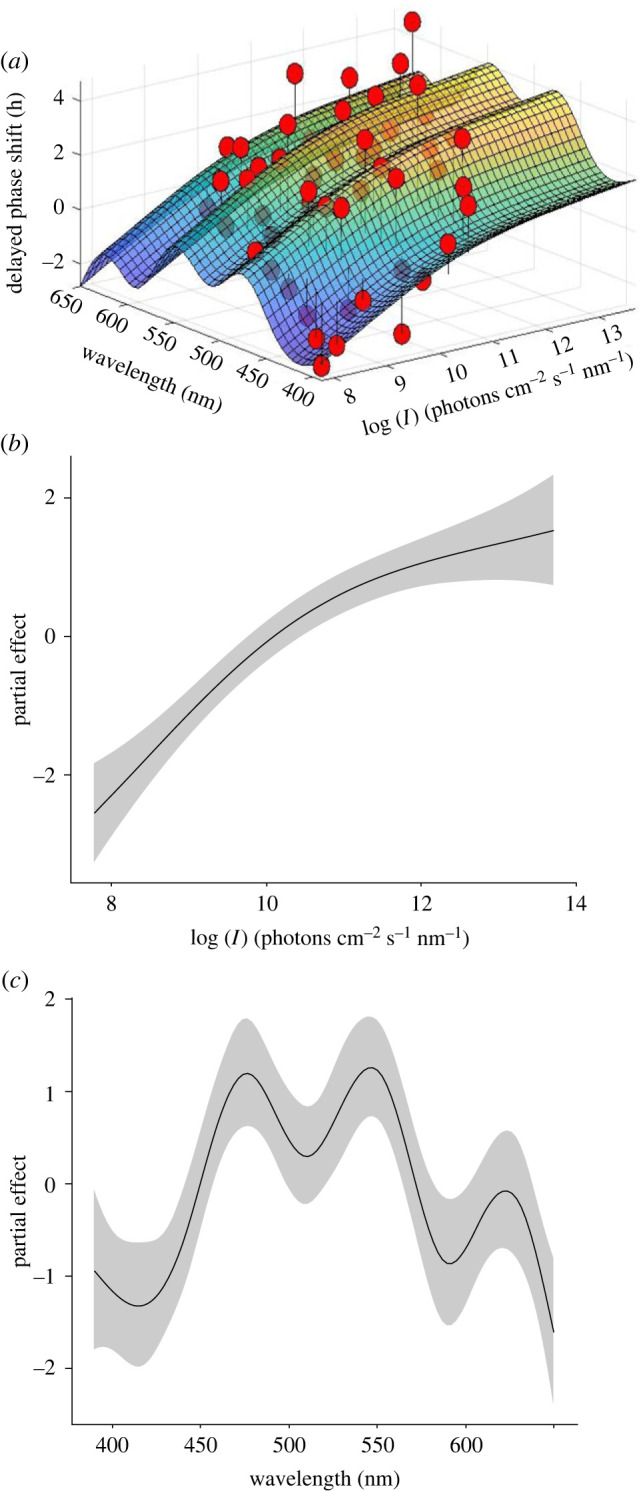
The action spectrum for circadian phase shift in response to different light intensities and wavelengths. (*a*) Three-dimensional model of the relationship between light intensity, wavelength and phase shift. Red symbols: averaged data of figure 2. (*b*) The partial effect on delayed phase shift response as a function of light intensity. (c) The partial effect on delayed phase shift response as a function of wavelength. Delayed phase shift is used in the analysis since the majority of the phase shift responses were delayed.

To analyse the effects of light intensity and wavelength on the phase shift responses, without any pre-assumptions of the data, we analysed all data (*n* = 1174, figure 2) with a GAM. Figure 3*a* shows the average phase shift data of figure 2 for the tested wavelengths and light intensities together with the three-dimensional fit following from the GAM modelling. The shape of the multi-modal relation between wavelength and phase shift did not essentially change from low to high light intensity (figure 3*a*, *p* = 0.44). The light intensity and phase shift were positively correlated, that is, higher light intensities induced stronger phase shifts in *Nasonia* (figure 3*b*, *p* < 0.001). Sensitivity peak wavelengths occurred at approximately 470 nm, approximately 540 nm and approximately 620 nm (figure 3*c*, *p* < 0.001).

### (b) Spectral sensitivity of the photoreceptors

**Figure 4.**
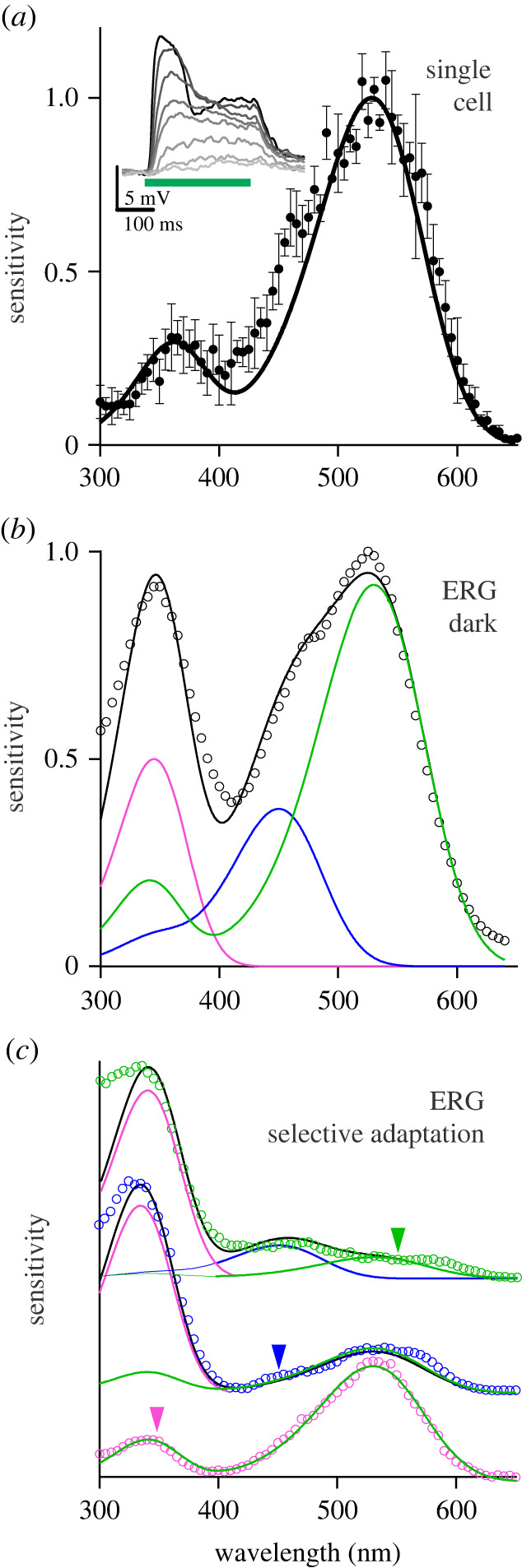
Spectral sensitivity of photoreceptors in the compound eyes. (*a*) Spectral sensitivity measured by intracellular recordings from the green-sensitive receptors in the compound eye (symbols; *n* = 3, mean ± s.d.), fitted with a rhodopsin template (solid curve, peak wavelength 528 nm); inset, voltage traces in response to 530 nm pulses, with relative intensity graded between 10^−2.4^ and 10^0^ in 10^0.4^ increments. (*b*) Spectral sensitivity of a dark-adapted compound eye measured by ERG (symbols), fitted with the sum of three rhodopsin templates (black curve) with peak wavelengths 340 nm (pink curve), 450 nm (blue curve) and 528 nm (green curve). (*c*) Spectral sensitivity (symbols) was measured by ERG recordings of the compound eye, selectively adapted with UV (365 nm, pink), blue (450 nm, blue) and green (535 nm, green); adapting wavelengths indicated by arrow heads (measured spectra are vertically shifted for clarity). The curves are weighted sums (black) of rhodopsin templates (pink, blue and green) with peak wavelength as in (*b*).

In order to reveal the photoreceptors involved in the circadian photoentrainment, we performed electrophysiological recordings in the compound eyes and ocelli. Intracellular recordings in the compound eyes with sharp microelectrodes yielded that the major photoreceptor class in the retina had a maximal sensitivity in the green wavelength range, at 528 nm (figure 4*a*). Consistent with the intracellular recordings, the spectral sensitivity of dark-adapted compound eyes determined via ERG recordings had a prominent peak in the green, at approximately 530 nm (figure 4*b*).

Since an ERG measures the response to light of the whole photosensitive organ, we proceeded with a series of selective adaptation experiments by applying saturating light of a specific wavelength, to suppress the sensitivity of particular photoreceptors. Applying selective adaptation to constant UV (365 nm), blue (450 nm) and green (535 nm) light yielded the sensitivity spectra of figure 4*c*. Using suitable weighting factors, the resulting spectra could be well fitted with the sum of three rhodopsin templates, with peak wavelength 340 nm, 450 nm and 530 nm, respectively (figure 4*b,c*).

**Figure 5.**
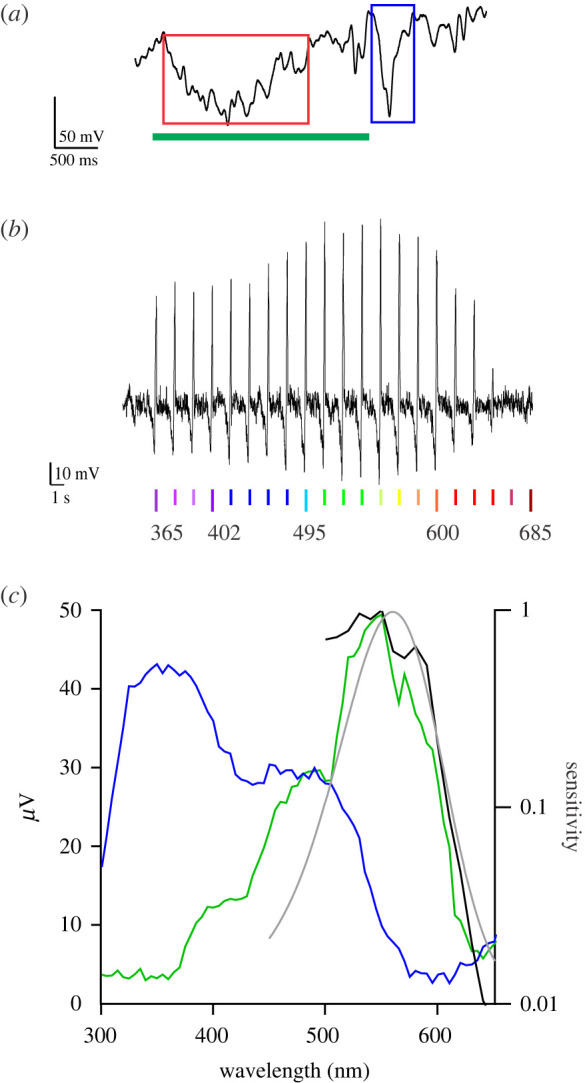
Spectral sensitivity of photoreceptors in the ocelli. (*a*) Response of the median ocellus to a 500 nm flash (duration: bottom green line) with a tonic (red box) and phasic (blue box) component. (*b*) ERG responses of the median ocellus stimulated with a spectral sequence of isoquantal flashes from red to UV. The responses are displayed time-reversed (i.e. from 365 to 685 nm). (*c*) Spectral characteristics of the median ocellus; left ordinate, spectral efficiency measured via the amplitude of the tonic (green curve) and phasic (blue curve) components; right ordinate, spectral sensitivity (black curve), derived from (*a*) with an intensity–response function, fitted with a rhodopsin template (grey line, peak wavelength 560 nm).

ERG recordings in the ocelli revealed a complex response to light stimuli, composed of a tonic phase during stimulation and a phasic off-response after the end of the flash (figure 5*a*). The ocelli of *Nasonia* are very fragile and were frequently damaged by the electrode, when penetrating the adjacent cuticle. The damage was in some instances selective, affecting only the structures that contribute the phasic or tonic response (possibly the photoreceptors and the interneurons, respectively). The spectral sensitivity of the ocelli measured via the phasic off-responses had distinct peaks in the UV and blue wavelength ranges, at approximately 360 nm and approximately 450–480 nm, respectively (figure 5*b*). The spectral sensitivity of the ocelli measured via the tonic responses peaked at approximately 550 nm, but it extended into the red part of the spectrum with possibly an additional peak at 580–620 nm (figure 5*c*; see also electronic supplementary material, SII).

### (c) Circadian entrainment is regulated by multiple photoreceptors

**Figure 6.**
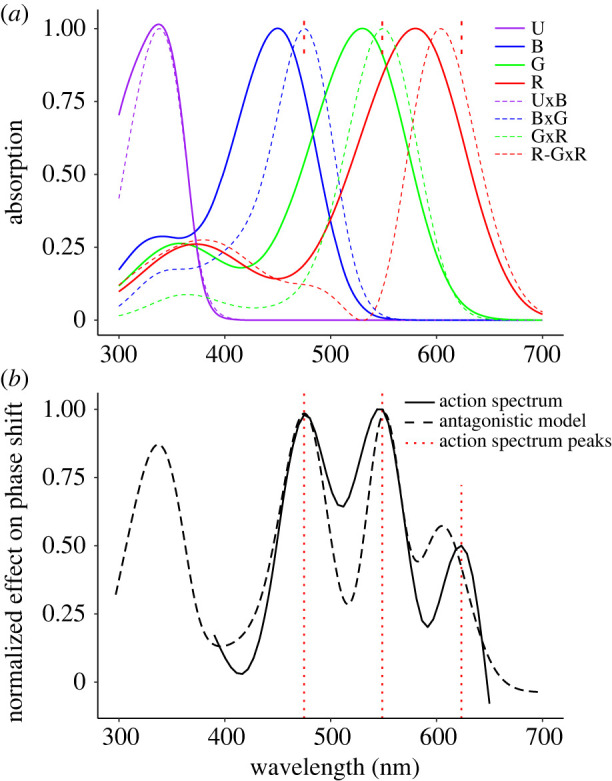
Multiple photopigments are involved in the circadian light entrainment of *N. vitripennis*. (*a*) Absorption spectra of the UV, blue and green visual pigments in the compound eye (and/or in the ocelli) and the ocellar red visual pigment (solid lines), and spectral sensitivities resulting from a heuristic model of antagonistic interactions between pairs of spectral photoreceptors with adjacent sensitivity peaks (dashed lines). (*b*) Circadian action spectrum (solid line, from figure 3*c*) and the weighted sum of antagonistic interactions between pairs of spectral photoreceptors from (*a*) (dashed line). The vertical red dotted lines in (*a*,*b*) indicate the peak wavelengths of the action spectrum.

To explain the action spectrum of the photoentrainment with the spectral sensitivities of the photoreceptors in the visual system of *Nasonia*, we compared the normalized circadian action spectrum with normalized rhodopsin absorption spectra. We assumed that the compound eye and ocelli possess the same UV, blue (B) and green (G) rhodopsins, furthermore that the ocelli have additional red (R) receptors, and that these photoreceptors provide input to the circadian entrainment system. However, the bands of the rhodopsin absorption spectra (peaking at 340, 450 and 530 nm) distinctly deviate from the spectral bands in the circadian action spectrum, peaking at 470 and 540 nm. The latter values rather coincide with the intersects of the blue and green rhodopsin spectra and of the green and red rhodopsin spectra, respectively (figure 6). This suggests that the action spectrum in the blue and green wavelength range is due to antagonistic interactions between different spectral channels in the retina of the compound eyes and/or of the ocelli. Similarly, the peak at approximately 600 nm could be caused by an antagonistic interaction of the red receptors in the ocelli and the green receptors in the compound eyes and/or ocelli.

To gain some insight, we developed a simple heuristic model of antagonistic interactions, based on the products of rhodopsin absorption spectra with adjacent peak wavelengths. With the antagonistic interactions of a blue and green receptor and of a green and red receptor, we could explain the spectral sensitivities peaking at 470 nm and 540 nm, respectively (figure 6*a*). Assuming the inhibition of a red receptor by the product of a green and red receptor can explain a sensitivity peak at approximately 600 nm (figure 6*a*). The calculated sensitivity spectra were further modified to yield narrower spectral peaks by calculating their exponentials, multiplied by linear weights and finally summated (figure 6*b*). The exponential factors and linear weights were chosen so that the final sum resembled the action spectrum of figure 3*c* (electronic supplementary material, SIII).

## 4. Discussion

To identify the circadian photoreceptors of the jewel wasp *Nasonia vitripennis*, we measured an action spectrum for the light-induced circadian phase shift and also the spectral sensitivity of the photoreceptors in the compound eyes and ocelli. The behavioural and electrophysiological experiments suggest the involvement in the photoentrainment of at least three rhodopsin classes, the blue, green and red receptors. The action spectrum could be understood as resulting from antagonistic interactions between the spectral channels in the retina of the compound eye and/or the ocelli. Although the action spectrum could not be determined at wavelengths below 390 nm, due to technical limitations, the local minimum in the action spectrum at approximately 410 nm (figure 3*c*) suggests the potential involvement of a UV-sensitive photoreceptor (modelled in figure 6*b*).

### (a) Opsin gene expression and electrophysiological measurements

Genomic analysis demonstrated that *Nasonia vitripennis* possesses four opsins, belonging to a UV, blue and long-wavelength (LW) rhodopsin, and also a fourth potential rhodopsin [28]. Their expression pattern was characterized via transcriptional profiling. Cycling expression patterns were found for all four opsins with a trough in the morning and a peak in the evening [39]. Our electrophysiological recordings indicate that the compound eyes of *Nasonia* have the typical substrate for trichromatic colour vision. The photoreceptors in the compound eye of *Nasonia* were estimated as UV (peak wavelength 340 nm), blue (450 nm) and green (530 nm). Those values are similar to the absorption peak wavelengths of the visual pigments in the majority of other hymenopteran insects, ranging from 324 nm to 370 nm for UV photoreceptors, 426 nm to 470 nm for blue photoreceptors and 512 nm to 553 nm for green photoreceptors [17,40]. Similar spectral sensitivities were observed in the ocelli, but interestingly the ocellar LW receptor appears to peak at a longer wavelength than the compound eye LW receptor. The extended sensitivity into the red region of the spectrum, with a peak wavelength at 560–580 nm of the *Nasonia* ocelli, could be due to the fourth opsin gene. Possibly, the ocelli express also the same green rhodopsin, peaking at 530 nm, that is present in the compound eye. In honeybees (*Apis mellifera*), the ocelli contain UV, blue and green-sensitive photoreceptors, but the latter's peak sensitivity is at approximately 500 nm. This indicates that the ocellar LW opsin differs from the LW opsin expressed in the compound eye [41]. Furthermore, we cannot rule out potential extra-retinal photoreceptors existing elsewhere in the central nervous system of *Nasonia*, since nothing is known about extra-retinal photoreception in Hymenoptera, and *Nasonia* lacks both a light-sensitive CRY and a honeybee *pteropsin*-like gene as potential neuronal photoreceptors.

### (b) The role of rhodopsins in the circadian phase shift response

The involvement of multiple rhodopsins in photoentrainment of the circadian phase shift response was confirmed by constructing a circadian action spectrum. The wavelength-dependent circadian photoentrainment follows a nonlinear pattern in *Nasonia*, where two major peaks correspond with the intersect of the absorption spectra of the blue and green photoreceptors and that of the green and red photoreceptor spectra, respectively. Strong phase shifts can also be triggered by red light (figure 2), yielding a peak at approximately 620 nm in the circadian action spectrum.

In *Drosophila*, all of the photoreceptors in the compound eyes, ocelli and extra-retinal photoreceptors may contribute to photic entrainment [2,42]. Our results indicate the involvement of both the compound eye and ocelli in circadian light entrainment in *Nasonia*. A similar function of the compound eye in circadian photoentrainment was also shown in cockroaches and crickets. In the cricket *G. bimaculatus*, especially the green-sensitive photoreceptor cells in the compound eyes were found as the major photoreceptors [7,11]. By contrast, in both cockroaches and crickets, ocelli appeared not to play a role in circadian photoentrainment, as only lesions of the compound eyes resulted in an impaired photoentrainment [8–10]. However, in some cricket species, the ocelli indirectly regulate the circadian rhythms by modulating the light sensitivity of the compound eyes [13].

The here obtained circadian action spectrum deviates from a simple sum of visual pigment spectra. Rather, the action spectrum's peak wavelengths coincide with the intersects of the rhodopsin spectra. This indicates that the neural pathway driving the entrainment is downstream of an antagonistic circuitry that processes the signals from the spectral photoreceptors, not dissimilar as occurs in visual colour processing. In *Drosophila* and other insects, colour vision starts with direct opponent synaptic interactions between the photoreceptors [43–45], involves colour-opponent neurons [46] as well as higher-order neurons in the brain with sharp spectral tuning [47], yielding spectral peaks similar to those in *Nasonia*'s action spectrum. In *Drosophila*, both visual and neuronal photoreceptors contribute to circadian entrainment: different photoreceptors can detect different intensities, wavelengths and durations of light information, and convey the light signals to the clock neurons via different mechanisms [15]. A colour-opponent input to the circadian rhythm is probably well suited for measuring the changes in the environmental colours, associated with the transitions between dawn, daylight and dusk. Moreover, it has been shown that vertebrates are able to detect changes in ambient spectral compositions of light during twilight but also in relation to the degree of overcast [48–50]. These colour-opponent signalling mechanisms result in more robust circadian entrainment and are involved in the regulation of phase shift and melatonin suppression in many vertebrates including humans [51–54].

In summary, we characterized four photoreceptor classes in the compound eye and ocelli of *Nasonia*. Our current circadian action spectrum suggests the participation of at least three rhodopsins (blue, green and red). Possibly, additional neuronal photoreceptors exist in *Nasonia* and contribute to circadian photoreception, but this requires additional investigations, for instance with knockdown/knockout of the photoreceptor genes expressed in the compound eyes and ocelli. The molecular mechanisms underlying the circadian light input pathway will be elucidated by gene expression analysis and functional genetics experiments.

## Data Availability

Data and scripts for analysis are available from the Dryad Digital Repository: https://doi.org/10.5061/dryad.bk3j9kdgk [55]. The data are provided in the electronic supplementary material [56].
